# Oil price uncertainty and corporate carbon performance: An international investigation

**DOI:** 10.1016/j.heliyon.2024.e36636

**Published:** 2024-08-21

**Authors:** Jibriel Elsayih, Rina Datt, Etaib E.E. Abdalmajeed

**Affiliations:** a Department of Accounting, Faculty of Economics, Site University, Sirte, 674, Libya; b School of Business, Western Sydney University, Parramatta, New South Wales, 2150, Australia

**Keywords:** Oil price uncertainty, Carbon performance, Greenhouse gas (GHG) emissions, Carbon accounting, Climate change

## Abstract

Drawing on legitimacy theory and stakeholder theory, this study examines whether oil price uncertainty (OPU) affects corporate carbon performance (CCP) in the international context. Based on data extracted from CDP (previously known as the Carbon Disclosure Project), World Bank, and Thomson Reuters Eikon databases, the study's sample consists of 9074 firm-year observations over the period 2011–2018 for all non-financial multinational companies invited to take part in the CDP questionnaire. Using an ordinary least squares regression model, we identify a strong relationship between OPU and carbon emissions performance. Our findings are robust to a battery of sensitivity tests, all of which support our original results. This study contributes new knowledge regarding the influence of OPU on CCP. The results will be of interest to investors and policymakers as they provide a useful basis for understanding OPU and its impact on CCP to promote better decision-making.

## 1 Introduction

Over the past decade, many studies have indicated that the problem of global warming caused by greenhouse gas (GHG) emissions will have significant impacts on the business sector, society, and ecosystems [[1], [2], [3]], as well as being a central concern for many international entities. Emissions of GHGs, including carbon dioxide (CO_2_) emissions, play a vital role in environmental degradation, as they are the main cause of the increasing global temperature [[4], [5], [6]]. At the beginning of the 1990s, environmental degradation and global warming began to attract global interest and were discussed, for example, at the Global Conference in Brazil in 1992 [7]. At this point, discussion began concerning the reduction of CO_2_ emissions, as this is key to addressing global warming [8], and this was later (in 2016) included in the Paris Agreement policy guidelines for the reduction of GHG emissions [4]. The United Nations Framework Convention on Climate Change (UNFCC) brought additional global attention to the matter when it announced that the goal for global warming should be to restrict it to below 1.5 °C above pre-industrial levels [9]. In addition, ref. [10] report, which provides the most comprehensive assessment of climate change to date, states that many countries around the world have signaled intentions to achieve net-zero emissions by 2050. Therefore, GHG emissions have become a critical issue for decision-makers in such countries.

According to British Petroleum, global energy demand will increase significantly by around a third over the next two decades [11]. Thus, world oil demand is expected to more than double by 2040 [12]. So companies would be expected to become more efficient. That might include better carbon control and thus we may expect such companies to come up with initiatives to better control carbon. Furthermore, in light of the increased demand for oil, governments, companies, and people may become more concerned than ever about the effects of oil prices on future GHG emissions [11]. Although energy and extractive industries are among the main pillars of economic development, they are major producers of GHGs [8]. In this regard, despite the introduction of various types of clean energy, crude oil remains the dominant energy source [13]. It is also a main source of gas flaring [14]. Hence, reliance on crude is a major environmental problem due to the CO_2_ emissions it produces [15]. At the same time, however, oil is an essential, high-demand commodity, making it critically important for economic activity around the world [[16], [17], [18], [19]]. Moreover, according to Ref. [20], crude oil prices largely determine energy demand, which in turn has a significant impact on carbon emissions. Therefore, when focusing on carbon emissions to address global climate change, it is important to consider the associations among crude oil prices, economic growth, and carbon emissions.

Several studies have investigated the impacts of oil prices on climate change. For instance, Ref. [21] study the impacts of crude oil prices on CO_2_ emissions, concluding that increased prices lead to increased consumption, which in turn harms the quality of the environment by emitting more carbon emissions. Ref. [8] reveals that oil price shock has a large impact on the level of carbon emissions in the short term but a weak effect in the long term. Ref. [22] suggested that short-term price increases boost carbon emissions while long-term ones have the opposite effect. Slightly more recently, ref. [23] concluded that higher oil prices result in significantly reduced CO_2_ emissions in the context of South Africa. Ref. [16] revealed that greater oil price volatility leads to better-quality carbon management systems. Ref. [11] specifically investigated the association between oil and natural gas prices and carbon efficiency and reported strong causality leading from oil/gas prices to carbon efficiency in the long run. However, no studies to date directly explore the relationship between oil price uncertainty (OPU) and corporate carbon performance (CCP), motivating us to examine this relationship. To bridge the knowledge gaps in the current literature, we examine whether OPU affects CCP at the international level. Although the literature documents a link between oil price fluctuations and GHG emissions, to the best of our knowledge, there are no reports of such a connection that considers carbon performance. By recognizing the importance of the impacts of OPU on CCP, we aim to address the following research question: does OPU affect CCP?

To investigate this, we use a sample of 9074 firm-year observations from all international companies that participated in the CDP (formerly known as the Carbon Disclosure Project) questionnaire over the period 2011–2018. Using an ordinary least squares (OLS) regression model, we identify a strong relationship between the OPU and carbon emissions performance. Our findings are robust to a battery of sensitivity tests, including Heckman's two-stage model, an alternative proxy of carbon performance and model specification, and subsample analysis. This study contributes new knowledge of the influence of OPU on CCP.

The main contributions of this study follow. First, the study expands evidence of a link between OPU and climate change by examining OPU and its effect on CCP. Second, to the best of our knowledge, this study is among the first to elucidate the impact of OPU on CCP. Unlike most previous studies on this topic [3,13,16,17,24], which generally focus on climate change, we consider CCP and how it is affected by OPU. Third, we use global data from the CDP database, which contains more credible and comprehensive information related to carbon than other sources that use general guidelines that all companies have to follow when reporting carbon emissions data. Fourth, we adopt legitimacy theory and stakeholder theory to design and interpret our empirical results, and thus our study promotes the validity and applicability of these theories for examining the impact of OPU on CCP from multiple perspectives. In addition, this paper adds to the current body of research by providing a more comprehensive understanding of this relationship that goes beyond the country level to the international level. Finally, our results provide new insights for policymakers that can be employed when evaluating current carbon policies and could help them develop appropriate regulations regarding carbon-related activity.

The remainder of this study is arranged as follows. Section 2 provides an overview of the theoretical frameworks employed. In Section 3, we present a literature review and develop the hypotheses. Section 4 discusses the research methodology, and Section 5 presents the results and discussion. Finally, Section 6 highlights our conclusions.

## 2 Theoretical framework

Legitimacy and stakeholder theories are two dominant theoretical frameworks in the field of environmental accounting, which addresses issues that impact the environment such as GHG emissions [[25], [26], [27], [28], [29], [30], [31]]. Both theories can be used to explain the motivations for companies' responses to public pressure and government policy regarding the disclosing of information on their environmental impacts [32]. A manager's primary concern is to meet the expectations and demands of the enterprise's stakeholders [33]; balancing disagreement with stakeholders to accomplish the company's goals is a manager's main task. Providing information on GHG emissions that they expect will satisfy stakeholder needs would be included in this [25].

Ref. [34] described legitimacy as “a condition or status which exists when an entity's value system is congruent with the value system of the larger social system of which the entity is a part. When a disparity, actual or potential, exists between the two value systems, there is a threat to the entity's legitimacy” (p. 122). Legitimacy theory is founded on the social contract that links the company to the society in which it operates [[35], [36], [37]]. From this perspective, to legitimize their activities and existence, companies make thorough disclosures of necessary carbon information [38]. Companies that provide positive information appear environmentally responsible. This appearance is bolstered by carbon emissions reductions that are in line with public expectations. In this way, carbon emissions reduction is used by companies to justify their operations [39]. In contrast, stakeholder theory highlights that, in the context of our research, OPU impacts CCP, which may influence firms directly or indirectly, favorably or unfavorably. Therefore, firms must address these issues in combating the future development of their entities. Any decisions made regarding these issues would have diverse social and environmental impacts and ultimately would affect various stakeholders. Moreover, ref. [40] further elaborate that the task of managers is to create value for stakeholders and ethical and all business decisions as one in value creation. Thus, the number of institutional investors in CDP respondents supports the validity of the stakeholder theory as CDP is a valuable platform for disclosing voluntary disclosure and carbon information [41]. Based on these considerations, firms are expected to disclose more information, such as carbon performance, when they seek to manage their relationships with influential stakeholder groups and thus enhance value creation for stakeholders [40,42].

## 3 Literature review and hypothesis development

GHG emissions control is a pillar of sustainable development [43], and companies are facing increasing pressure to minimize their emissions [44,45]. At present, crude oil, the burning of which produces carbon emissions, is the main source of energy consumed by companies and has become closely related to environmental performance [13]. A link has appeared between crude oil prices and the consumption of fossil fuels, which produce approximately 40 % of global carbon emissions [4]. In addition, both carbon emissions and crude oil prices have attracted significant public interest in recent years [24,46].

Oil prices have a powerful influence on various economic variables including gross domestic product, investments, income levels, trade balance, and carbon emissions, among others [[47], [48], [49], [50], [51], [52], [53]]. Ref. [54] used a sample of 5740 firms across eight countries to investigate the nexus between carbon emissions and human capital efficiency and report a negative relationship between investment in human capital and carbon emissions. They suggest that the relevance of firm-level decisions in limiting carbon emissions is ignored. In the Chinese setting, ref. [55] examine whether the international oil price uncertainty affects corporate investment expenditures. They find that oil price uncertainty has a significantly negative impact on corporate investment expenditures. Ref. [13] investigated the impacts of fluctuations in international crude oil prices and corporate development levels on the carbon emissions of 1089 firms and found that an increase in OPU can inhibit carbon emissions but that this is also influenced by specific firm characteristics such as whether or not it is state-owned. In a similar setting, ref. [56] use firm-level data to study the influences of three classical oil price shocks (oil supply shocks, oil aggregate demand shocks, and oil-specific demand shocks) on corporate firms in China. They report that both oil aggregate demand and specific demand shocks harm corporations, while oil supply shocks have the opposite effect. In addition, firms in energy-intensive sectors are more sensitive to such shocks. Therefore, a spike in oil prices, one such shock, could in theory lower corporate carbon emissions, reduce per-capita consumption of oil, and force firms, in China and throughout the world, to become cleaner and to consume less carbon. This in turn would promote a net-zero-carbon society [57]. At the same time, ref. [23] find that, in South Africa, renewable energy improves environmental quality in the short and long term but that a shift to such energy can lead to less oil demand and thus a spike in oil prices. Considering that South Africa is an oil-importing country, this trend is compounded as global oil prices increase. However, an increase in price would in theory lead to still lower demand and thus fewer carbon emissions. To maintain this dynamic, the country could increase its interest rates, which should reduce investments. Ultimately, this could lead to lower energy usage among firms (and thus keep emissions down). Ref. [22] reported similar results in a study of Pakistan from 1971 to 2014 indicating that a spike in oil prices leads to a short-term increase in carbon emissions that eventually decline.

On the other hand, several studies conducted in the United States suggest that an increasing GDP per se does not lead to greater carbon emissions in the long run; rather, the types of energy used do, where more burning of fossil fuels (vs. the use of alternative energy sources) is directly associated with greater carbon emissions [52]. In addition, ref. [53] reports that, in the United States, energy use tends to increase as the GDP increases. Ref. [58] studied micro-level factors such as firm/customer dynamics and market behavior and reported that OPU has a distorted effect on investments and that this distortion is more evident in small companies than in larger firms. In addition, ref. [59] indicate that the quality of the environment is degraded by the CO_2_ emissions produced by high levels of oil consumption. However, an increase in oil prices can reduce environmental deterioration by reducing the use of oil products.

From the studies mentioned above, we argue that during periods of high economic growth, firms may reduce information asymmetry with external stakeholders. During such periods, the demand for products and services tends to be high and thus firms may need external funds to finance their operations to meet increasing demand. In this context, reducing information asymmetry through carbon disclosure, leading to a lower cost of financing. In addition, based on legitimacy theory, we predict that OPU positively impacts CCP, suggesting that firms implement carbon mitigation activities to legitimize themselves to ensure that they meet the demands of various stakeholders. Hence, it can be argued that fluctuating crude oil prices can impact environmental quality. More specifically, crude OPU can reduce the consumption of energy, thereby decreasing corporate carbon emissions. Furthermore, companies tend to improve their carbon emissions during periods of high economic growth because the incremental benefit of such improvement outweighs the costs. Hence, we expect a positive effect of OPU on CCP (i.e., lower GHG emissions). From this, the following hypothesis is proposed.

H1 Oil price uncertainty has a positive impact on corporate carbon performance.

## 4 Research design

### 4.1 Sample and data

Our original sample includes 46,904 international firm-year observations from companies affiliated with 49 countries that were invited by the CDP to answer its annual survey from 2011 to 2018. We chose this period because the CDP database is more complete and reliable starting in 2011 [60,61] and also to avoid the possible effects of global crises such as the COVID-19 pandemic and the Russia–Ukraine crisis, all of which significantly affected the prices of crude oil and natural gas [62]. Also, the CDP climate questionnaires become more consistent and comparable in format during this time period, thus minimizing the risk of missing values for any variables. We follow prior studies and do not include financial sector companies (3358 observations) in our sample due to differences in the nature of their reporting of their activities and environmental regulations [38]. Observations with missing or invalid data (totaling 34,472 observations) are removed from the sample. This yields a final sample of 9074 firm-year observations (Table 1). Carbon emissions data are obtained from the CDP annual questionnaire. OPU data are drawn from the World Bank Dataset. Finally, all financial data are either downloaded from the World Bank dataset or Thomson Reuters Eikon DataStream.

**Table 1:** Sample structure.

Original sample	Number of obs.
All companies invited by the CDP to answer its annual survey from 2011 to 2018.	46,904
Companies with incomplete carbon information, either direct emissions (Scope 1) or indirect emissions (Scope 2).	(32,233)
Companies in the financial sector.	(3358)
Companies with missing financial data from Thomson Reuters Eikon DataStream.	(2239)
**Final sample**	**9074**

### 4.2 Empirical model

We use the following empirical specification to estimate the association between OPU and carbon emissions performance:

(1) *CCP*_*it*_ = *β*_*0*_*+ β*_*1*_*OPU*_*it*_*+ β*_*2*_*SIZE*_*it*_*+ β*_*3*_*ROA*_*it*_*+ β*_*4*_*LEV*_*it*_*+ β*_*5*_*CAPINT*_*it*_*+β*_*6*_*TOBINQ*_*it*_*+ β*_*7*_*LnOPEN*_*it*_*+ β*_*8*_*LnGDP*_*it*_*+ β*_*9*_*WGI*_*it*_*+ β*_*10*_*ETS*_*it*_ + *Sector Effect* + *Year Effect* + *Country Effect* +_*Єit*_

Here, the dependent variable CCP represents carbon performance. OPU, an independent variable, is the main variable of interest, and the other variables, namely, *Ln*TCI, *SIZE*, *ROA*, *LEV*, *CAPINT*, *TOBINQ*, *LnOPEN*, *LnGDP*, *WGI*, and *ETS*, are control variables. For a detailed description of all of the variables please see Appendix 1.

### 4.3 Measurement of variables

#### 4.3.1 Dependent variable (CCP)

We follow previous studies [26,38,63,64] and use the total carbon emissions intensity as an overall indicator of carbon performance, calculated as the natural logarithm of the ratio of total scope 1 (direct) and scope 2 (indirect) emissions to total sales at the end of the fiscal year.

#### 4.3.2 Independent variable (OPU)

OPU is calculated using the standard deviation of daily returns for crude oil prices [16,65].

#### 4.3.3 Control variables

Based on prior literature [60,[66], [67], [68], [69]] among others, we include a battery of control variables in our model that are known to affect the variables of concern. As a first step, we control for firm size (SIZE) using the natural logarithm of market capitalization [68]. Moreover, profitability (ROA) and leverage (LEV) are controlled. The first one is measured by the net income before extraordinary items/preferred dividends, divided by total assets, and the second variable is calculated by long-term debt divided by total assets at the end of the fiscal year [67,69]. We also control for capital intensity (CAPINT) utilizing the ratio of property, plant, and equipment (PPE) divided by total assets as per ref. [66]. Another control variable is Growth opportunity (TOBINQ), which is measured as the market value of common equity divided by the book value of total assets [70]. LnOPEN and LnGDP are controlled for as additional firm-specific variables. We measure LnOPEN as the natural logarithm of the sum of exports and imports divided by GDP and multiplied by 100 [44], and LnGDP is calculated as the natural logarithm of real gross domestic product per capita [68]. Further, we include WGI and ETS as control variables. We capture the first variable by using scores for factors derived from factor analyses of six dimensions of the World Bank world governance indicators and calculate the second one as a dummy variable that equals 1 if the firm is located in a country with an operating national emissions trading scheme and 0 otherwise [39,66]. Finally, sector and year fixed effects are also controlled for in the models.

## 5 Results

### 5.1 Descriptive statistics

Table 2 presents the descriptive statistics for the independent and control variables involved in our model. The mean (median) value of CCP is −2.661 (−2.938), with a standard deviation of 1.893. The respective values for OPU are 0.020 and 0.017. The control variables show a very similar pattern, in that the means are not far from the medians. These results align with those of previous research [44,65,66].

**Table 2:** Descriptive statistics.

Variable	Obs.	Mean	Median	Std. Dev.	Min	Max
**CCP**	9074	−2.661	−2.938	1.893	−7.090	2.061
**OPU**	9074	0.020	0.017	0.006	0.011	0.031
**SIZE**	9074	15.657	15.588	1.651	11.864	20.673
**ROA**	9074	0.045	0.041	0.060	−0.167	0.227
**LEV**	9074	0.194	0.180	0.144	0	2.821
**CAPINT**	9074	0.318	0.270	0.224	0.008	0.897
**TOBINQ**	9074	2.143	3.106	2.061	−3.507	4.652
**LnOPEN**	9074	3.995	4.085	0.511	3.199	5.421
**LnGDP**	9074	10.256	10.647	1.194	3.088	11.409
**WGI**	9074	1.127	1.341	0.691	−1.507	1.852
**ETS**	9074	0.294	0	0.456	0	1

Note: Descriptions and sources of all variables are reported in Appendix 1.

Table 3 presents the Pearson correlation matrix among the regression variables. We find that CCP has a statistically significant relationship with ROA, LEV, CAPINT, LnOPEN, LnGDP, WGI, and ETS but a nonsignificant one with OPU, SIZE, and TOBINQ. In addition, the results do not appear to show a serious threat of multi-collinearity because of the low correlation coefficients present in the data series. Following the suggestion of ref. [71], we find that all correlation coefficients in our study are below Pearson's correlation rule of thumb (with an upper bound of 0.551).

**Table 3:** Correlation matrix.

Variable	CCP	OPU	SIZE	ROA	LEV	CAPINT	TOBINQ	LnOPEN	LnGDP	WGI	ETS
**CCP**	1.000										
**OPU**	−0.006	1.000									
**SIZE**	−0.006	0.131***	1.000								
**ROA**	−0.193***	−0.007	0.231***	1.000							
**LEV**	0.231***	−0.01	0.031***	−0.206***	1.000						
**CAPINT**	0.616***	−0.037***	−0.003	−0.149***	0.295***	1.000					
**TOBINQ**	−0.009	−0.024**	−0.022**	0.095***	0.016***	−0.023**	1.000				
**LnOPEN**	−0.026**	0.037***	−0.192***	0.002	−0.061***	−0.013	0.131***	1.000			
**LnGDP**	−0.137***	0.087***	0.114***	−0.037***	0.086***	−0.069***	0.248***	−0.090***	1.000		
**WGI**	−0.147***	0.036***	0.044***	−0.008	−0.014	−0.051***	0.399***	0.075***	0.551***	1.000	
**ETS**	0.188***	0.008	0.090***	−0.069***	0.063***	0.103***	0.036***	0.051***	0.074***	0.056***	1.000

Notes: *t* statistics are reported in parentheses; ***, **, and * denote significance at the 1 %, 5 %, and 10 % levels, respectively. Descriptions and sources of all variables are reported in Appendix 1.

### 5.2 Multivariate analyses

Table 4 reports the OLS results concerning the effects of OPU on CCP. In Column (1), we only include OPU, together with dummy variables for sector, year, and country. In Column (2), we show both OPU and all control variables. Our analysis is primarily based on the findings that we report in Column (2). The results of the F-test for the OLS regression models are statistically significant at the p < 0.01 level, indicating that the coefficients of OPU and other independent variables can explain the significant variation in CCP. In addition, the adjusted R2 (46.83 % and 58.26 % of the variance, respectively) suggests that OPU and other variables play a significant role in explaining variation in CCP. Moreover, to effectively detect outliers, we winsorize all continuous variables at the 10th and 90th percentiles.

**Table 4:** Results of OLS regression.

Variable	Dependent variable: CCP	
	Model (1) Without control variables	Model (2) Full sample
**OPU**	90.718*** (2.93)	88.581*** (2.80)
**SIZE**		0.020** (2.07)
**ROA**		−2.408*** (−9.90)
**LEV**		0.542*** (5.16)
**CAPINT**		3.212*** (42.45)
**TOBINQ**		0.011 (0.88)
**LnOPEN**		0.149 (0.95)
**LnGDP**		0.161** (2.32)
**WGI**		−0.129 (−0.48)
**ETS**		0.305*** (10.00)
**Constant**	−1.920*** (−2.93)	−6.526*** (−4.18)
**Observations**	9074	9074
**Sector FE**	Yes	Yes
**Year FE**	Yes	Yes
**Country FE**	Yes	Yes
**Adjusted R**^**2**^	0.4683	0.5826
**F- value**	127.87	176.89
**Prob > F**	0.000	0.000

Notes: *t* statistics are reported in parentheses; ***; **, and *denote significance at the 1 %, 5 %, and 10 % levels, respectively. We winsorize most of our variables at the 10th and 90th percentiles. Descriptions and sources of all variables are reported in Appendix 1.

The OLS regression estimates shown in Column (1) of Table 4 indicate that the regression coefficient of OPU is positively associated with CCP at the 1 % significance level, which strongly supports our hypothesis. This positive effect implies that increasing OPU may lead to better carbon performance in terms of lower carbon emissions. This finding is in line with the view of Ref. [13], who contend that an increase in international crude OPU could inhibit carbon emissions as firms adopt proactive environmental strategies to improve carbon performance by, for example, launching detailed initiatives to reduce carbon emissions [26,72]. A possible explanation for this positive relationship is that both the Kyoto Protocol and the Paris Agreement prescribe changes in carbon emissions using many policy instruments, which may have also had an impact on the market price of crude oil, thereby improving carbon performance. These findings are in line with ref. [13] and ref. [16], who indicate that firms tend to improve their carbon emissions during periods of high OPU to heighten investor confidence. The results are also similar to those reported by Ref. [11], who show that increasing crude oil prices can increase carbon efficiency by reducing CO_2_ emissions. However, the results contradict ref. [23,73,74], who suggest that oil prices are inversely related to emissions.

In terms of the control variables, the coefficient of SIZE has a positive and significant relationship with CCP, which aligns with previous research [63]. This implies that large firms may be the main contributors to increased GHG emissions and may be more likely to engage in carbon reporting practices. In addition, the positive relationship between LEV and CCP is in line with the results of ref. [38] suggesting that highly leveraged companies are more exposed to increased GHG emissions. The OLS regression results also show a positive association between CAPINT and CCP, which is consistent with the results of ref. [66]. That is, companies with high capital expenditures tend to have a greater potential for mitigating climate change. This implies that such companies are willing to increase investment in CCP such as purchasing advanced machinery and equipment that is more sustainable and eco-friendly, which could result in reduced carbon emissions [75]. Furthermore, the coefficient for LnGDP has a positive relationship with CCP, in line with prior research [23]. ROA is negatively related to CCP, whereas TOBINQ, LnOPEN, and WGI exhibit no association with CCP, indicating that these factors do not play a significant role in carbon performance.

### 5.3 Sensitivity analysis

In the following sections, we present a battery of sensitivity tests to check the robustness of our main results.

#### 5.3.1 Self-selection bias

In Table 5, we report our estimates of the Heckman two-stage model [76] as a further robustness test. We perform this test to alleviate the endogeneity bias that exists in self-selected samples. To address this and control for this bias in our analyses, we follow prior work [16,39,77] among others, and conduct Heckman's two-stage tests. In the first step of this method, we formulate a probit regression model (see Appendix 1 for more details on the definitions of all variables) as given below to estimate the probability of selection:

(2) *CDis*_*it*_ = *β*_*0*_*+ β*_*1*_*OPU*_*it*_*+ β*_*2*_*SIZE*_*it*_*+ β*_*3*_*ROA*_*it*_*+ β*_*4*_*LEV*_*it*_*+ β*_*5*_*CDMean*_*it*_*+β*_*6*_*NEW*_*it*_*+ β*_*7*_*LAW*_*it*_ + *Sector Effect* + *Year Effect* + _*Єit*_

**Table 5:** Results of Heckman's two-stage model.

Variable	Step 1	Step 2
	Dependent variable: CDis	Dependent variable: CCP
**OPU**		151.160** (2.18)
**SIZE**	0.304*** (53.38)	−0.177*** (−6.59)
**ROA**	−0.070*** (−14.96)	3.073*** (3.16)
**LEV**	0.272*** (4.73)	0.283 (1.16)
**CAPINT**		3.15*** (24.38)
**TOBINQ**		−0.012*** (−9.84)
**LnOPEN**		0.648*** (2.60)
**LnGDP**		−0.009 (−0.08)
**WGI**		−0.343 (−0.65)
**ETS**		1.220*** (20.31)
**CDMean**	3.288**** (4.61)	
**NEW**	−0.470*** (−12.33)	
**LAW**	−0.243*** (−14.07)	
**Constant**	−5.990*** (−25.79)	13.181*** 4.67)
**Industry FE**	Yes	Yes
**Year FE**	Yes	Yes
**Country FE**	No	Yes
**Observations**	27,717	
**Uncensored**	7617	
**Wald chi**^**2**^**(Prob > chi**^**2**^**)**	5225.51(0.000)	

Notes: This table reports the results of Heckman's two-stage model. Column (1) shows the estimation results for the first stage. Column (2) displays the results of the second stage. *t* statistics are presented in parentheses; ***, **, and * denote significance at the 1 %, 5 %, and 10 % levels, respectively. We winsorize most of our variables at the 10th and 90th percentiles. The descriptions and sources of all variables are reported in Appendix 1.

In the second step, we re-estimate Equation (1), using the inverse Mills ratios created in the first step. In Column (1), we provide the estimation results for the first stage, while in Column (2), we present the estimation results for the second stage. We find that, after controlling for sampling bias, the results are still in the same direction as those shown in Table 4 and hence provide further support for our hypothesis.

#### 5.3.2 Instrumental variable test

To consolidate the robustness of our main analysis, we follow ref. [78] and ref. [79] and use an instrumental variable approach, utilizing the lagged value of OPU by two periods. The results reported in Column (1) of Table 6 show that the coefficient of the main variable is quite close to our original result presented in Table 4 in terms of significance, which bolsters the validity of our hypothesis.

**Table 6:** Robustness tests.

Variables	IV test	RE Model	Alternative Variables	
			Dependent	Independent
**OPU**		67.110*** (4.64)	119.345*** (2.14)	
**OPU1**				0.375*** (2.80)
**OPU**_**t-2**_	85.090*** (2.69)			
**Controls**	Yes	Yes	Yes	Yes
**Constant**	−6.294*** (−4.03)	−5.107*** (−7.08)	−6.297** (−2.28)	−6.297** (−2.28)
**Observations**	9074	9074	9074	9074
**Sector FE**	Yes	No	Yes	Yes
**Year FE**	Yes	Yes	Yes	Yes
**Country FE**	Yes	Yes	Yes	Yes
**R-squared (%)**	0.5853	0.3727	0.0248	0.5859
**Wald chi**^**2**^**(Prob > chi**^**2**^**)**		1004.04*** (0.000)		
**F-Value (Prob > F)**	176.41*** (0.000)		4.21*** (0.000)	176.89*** (0.000)

Notes: This table reports the results of three different robustness tests. Column (1) presents the results of the IV test. Column (2) presents those of using another specification model, while Columns (3) and (4) show those of using an alternative measure of dependent and independent variables, respectively. *t* statistics are reported in parentheses; ***, **, and * denote significance at the 1 %, 5 %, and 10 % levels, respectively. We winsorize most of our variables at the 10th and 90th percentiles. Descriptions and sources of all variables are reported in Appendix 1.

#### 5.3.3 Heterogeneity test

To control for possible unobserved firm-level heterogeneities, we re-estimate our regression utilizing an alternative approach, namely, a random-effects (RE) regression model. The results presented in Column (2) of Table 6 indicate that the regression coefficient for OPU is still positive and has the same sign and significance, similar to those reported in Table 4; this proves the validity of our expectations.

#### 5.3.4 Alternative measures

In this section, to check the reliability and validity of our main results, we employ two different measures of CCP and OPU. First, we use the natural logarithm of total carbon emissions scaled by total assets [80] as another way to calculate CCP and re-run the main model. Second, following ref. [81] and ref. [82], we use the global price of Brent crude (OPU1) as an alternative crude oil price to calculate OPU described in Section 4.3.1. The findings in Columns (3) and (4) of Table 6 suggest that both coefficients of OPU are still positive and statistically significant at a 1 % level, which is in the same direction as the results presented in Table 4. This implies that our findings are highly robust, even when we apply alternative measures of both variables. For the sake of brevity, we only show the coefficients for variables of interest. Overall, the robust estimation results provide significant support for our hypothesis.

#### 5.3.5 Subsample analysis effects

Another two steps are taken in this section to check the robustness of our results. First, we partition our respondent companies into two groups based on company size. Following ref. [83], the first subsamples are generated from larger companies that have a firm size greater than the average firm size of the full sample, whereas the second subsamples are created from smaller companies that are smaller than the average size for the total sample. We re-run Equation (1) separately for each group and find that the effect of OPU on CCP is more remarkable and more significant for larger companies than smaller ones. The results are presented in Columns (1) and (2) of Table 7. Second, because fluctuations in oil price may play different roles in the carbon sensitivity of different companies, we split the entire sample into two groups, one for carbon-intensive sectors (such as materials, energy, or utilities), and the other for less-carbon-intensive sectors (such as consumer staples, financial, consumer discretionary, health care, industrials, information technology, and telecommunications). As shown in Tables 7 and in the less-carbon-intensive sectors (Column 3), the estimation results have similar signs and significance patterns to those shown in Table 4. That is, OPU remains positively associated with CCP. However, in the carbon-intensive sectors (Column 4), the estimation results with OPU do not support our earlier results, which implies that the effect of OPU on CCP does not seem to play a vital role in the carbon-related activities of these sectors. Overall, our analysis provides evidence that OPU has important implications for firms that work in less-carbon-intensive sectors.

**Table 7:** Robustness test: the results of subsample effects.

Variable	DV: CCP			
	FSize = 1	FSize = 0	IntSector = 1	IntSector = 0
**OPU**	110.180*** (2.84)	59.307 (1.20)	22.224 (0.34)	105.819*** (3.04)
**Controls**	Yes	Yes	Yes	Yes
**Constant**	−9.829*** (−4.51)	−2.762 (−1.21)	−3.965*** (−1.21)	−9.346 *** (−5.42)
**Observations**	4537	4537	2628	6446
**Sector FE**	Yes	Yes	Yes	Yes
**Year FE**	Yes	Yes	Yes	Yes
**Country FE**	Yes	Yes	Yes	Yes
**Adjusted R**^**2**^	0.6576	0.5628	0.2577	0.3944
**F-value**	133.02	82.09	15.95	65.58
**Prob > F**	0.000	0.000	0.000	0.000

Notes: This table reports the relationship between OPU and CCP for subsample effects. Columns (1) and (2) provide results of the subsample of larger and smaller firms based on the median value of firm size. Columns (3) and (4) describe the results of the subsample based on the sector affiliation (carbon-intensive sectors vs. less-carbon-intensive sectors). *t* statistics are presented in parentheses; ***, **, and * denote significance at the 1 %, 5 %, and 10 % levels, respectively. We winsorize most of our variables at the 10th and 90th percentile. Descriptions and sources of all variables are reported in Appendix 1.

## 6 Conclusion

Although crude oil plays a key role in sustainable economic development, little research has addressed the effects of crude OPU on carbon performance. This study investigates the effects of OPU on CCP in the global context for the period of 2011–2018. Using OLS regression models, we find that OPU has a positive effect on CCP at the 1 % significance level, which is consistent with our expectations. This means that increasing OPU can enhance CCP by resulting in a decrease in crude oil use, thereby reducing carbon emissions. In addition, the results also suggest that carbon emissions performance is sensitive to some financial indicators, such as firm size, leverage, capital intensity, foreign sales, and economic growth.

These results have significant implications. First, our results may help policymakers develop policies and procedures related to improving carbon performance by using mitigation strategies that may affect OPU. They also can draw on the findings of this study when developing standards for reporting corporate climate change information and provide insights into the factors that can influence OPU. In addition, our results may provide corporate directors and decision-makers with key information to achieve carbon policy goals, where such goals may play an important role in the stabilization of oil prices. Furthermore, our findings could help company managers set up appropriate policies that may help minimize the impacts of OPU on carbon emissions. Moreover, our results could be of interest to investors, as they provide investors with a good understanding of the relationship between carbon performance and other variables, which could lead to appropriate investment decisions. Finally, the results are also a useful guide for those who establish accounting standards, other policymakers, and society in general regarding the determinants of carbon performance.

However, this study is not free of limitations, which should be highlighted and addressed in future research. First, the carbon data are taken from the CDP, so the results do not apply to information disclosed through different media, for instance, a firm's website or press releases. Second, this study incorporates companies that participated in the CDP survey for the period 2011–2018. This may lead to the results not being valid for generalization. In this regard, further research could be done to cover a longer period and other firms that do not take part in the CDP's climate change questionnaire. Third, we ignore the influence of the COVID-19 pandemic and the Russia–Ukraine crisis on the relationship between CCP and OPU, which may be a jumping-off point for future studies.

## Data availability statement

The data that support the findings of this study are available from the corresponding author.

## CRediT authorship contribution statement

**Jibriel Elsayih:** Writing – original draft, Supervision, Methodology. **Rina Datt:** Writing – review & editing. **Etaib E.E. Abdalmajeed:** Writing – review & editing, Resources.

## Declaration of competing interest

The authors declare that they have no known competing financial interests or personal relationships that could have appeared to influence the work reported in this paper.

## Figures and Tables

**Table:** 

Variable	Lebel	Description/reference	Source
Dependent variable			
Carbon emissions performance	CCP	Natural logarithm of total scope 1 (direct) and scope 2 (indirect) emissions divided by total sales in thousands of US dollars [26,38].	CDP
**Independent variable**			
Oil price uncertainty	OPU	Standard deviation of daily returns of crude oil price [16,65]	World Bank
**Control variables**			
Firm size	SIZE	Natural logarithm of market capitalization [68].	DataSream
Profitability	ROA	Net income before extraordinary items/preferred dividends divided by total assets at the end of the financial year [67].	DataSream
Leverage	LEV	Long-term debt divided by total assets at the end of the fiscal year [69].	DataStream
Capital intensity	CAPINT	Ratio of property, plant, and equipment (PPE) divided by total assets [66].	DataStream
Growth opportunity	TOBINQ	Market value of common equity divided by the book value of total assets [70].	DataStream
Openness of economy	LnOPEN	Natural logarithm of the sum of exports and imports divided by GDP and multiplied by 100 [44].	World Bank
Economic development	LnGDP	Natural logarithm of real gross domestic product per capita [68].	World Bank
World Governance Index	WGI	Scores for factors derived from factor analyses of six dimensions of the World Bank world governance indicators [66].	World Bank
Emissions trading scheme	ETS	A dummy variable that equals 1 if the firm is located in a country with an operating national emissions trading scheme and 0 otherwise [39].	https://icapcarbonaction.com/en
Carbon disclosure mean	CDMean	The mean industry disclosure score [60,68].	CDP
Newness of assets	NEW	Ratio of net PPE of firm i in year t to gross PPE [84].	DataStream
Legal system	LAW	A dummy variable equaling 1 if the firm is located in a civil/code law country and 0 otherwise [38].	Wikipedia (2020)

## Appendix 1

Variable definition/references and sources.

## References

[1] Shang Y., Han D., Gozgor G., Mahalik M.K., Sahoo B.K. (2022). The impact of climate policy uncertainty on renewable and non-renewable energy demand in the United States. Renew. Energy.

[2] Tanthanongsakkun S., Kyaw K., Treepongkaruna S., Jiraporn P. (2023). Carbon emissions, corporate governance, and hostile takeover threats. Bus. Strat. Environ..

[3] Ullah S., Chishti M.Z., Majeed M.T. (2020). The asymmetric effects of oil price changes on environmental pollution: evidence from the top ten carbon emitters. Environ. Sci. Pollut. Control Ser..

[4] Chaudhry I.S., Azali M., Faheem M., Ali S. (2020). Asymmetric dynamics of oil price and environmental degradation: evidence from Pakistan. Review of Economics and Development Studies.

[5] Stocker T.F., Qin D., Plattner G.-K., Tignor M.M., Allen S.K., Boschung J., Nauels A., Xia Y., Bex V., Midgley P.M. (2014).

[6] Usman O., Olanipekun I.O., Iorember P.T., Abu-Goodman M. (2020). Modelling environmental degradation in South Africa: the effects of energy consumption, democracy, and globalization using innovation accounting tests. Environ. Sci. Pollut. Control Ser..

[7] United-Nations (3-14 June 1992). Report of the United Nations Conference on Environment and Development, Rio de Janeiro. https://www.un.org/en/conferences/environment/rio1992.

[8] Zou X. (2018). VECM model analysis of carbon emissions, GDP, and international crude oil prices. Discrete Dynam Nat. Soc..

[9] (2019). UNFCC. Cut global emissions by 7.6 percent every year for next decade to meet 1.5°C Paris target - UN report. Available.

[10] Shukla P.R., Skea J., Slade R., Al Khourdajie A., van Diemen R., McCollum D., Pathak M., Some S., Vyas P., Fradera R., Belkacemi M., Hasija A., Lisboa G., Luz S., Malley J. (2022). IPCC: Climate Change, Mitigation of Climate Change. Contribution of Working Group III to the Sixth Assessment Report of the Intergovernmental Panel on Climate Change.

[11] Liu H., Pata U.K., Zafar M.W., Kartal M.T., Karlilar S., Caglar A.E. (2023). Do oil and natural gas prices affect carbon efficiency? Daily evidence from China by wavelet transform-based approaches. Resour. Pol..

[12] Broadstock D.C., Filis G. (2014). Oil price shocks and stock market returns: new evidence from the United States and China. J. Int. Financ. Mark. Inst. Money.

[13] Wei P., Li Y., Ren X., Duan K. (2022). Crude oil price uncertainty and corporate carbon emissions. Environ. Sci. Pollut. Control Ser..

[14] Edino M.O., Nsofor G.N., Bombom L.S. (2010). Perceptions and attitudes towards gas flaring in the Niger Delta, Nigeria. Environmentalist.

[15] Eltaib E.E. (2012).

[16] Bugshan A., Elsayih J. (2023). Oil price uncertainty and carbon management system quality. Econ. Lett..

[17] Agbanike T.F., Nwani C., Uwazie U.I., Anochiwa L.I., Onoja T.-G.C., Ogbonnaya I.O. (2019). Oil price, energy consumption and carbon dioxide (CO 2) emissions: insight into sustainability challenges in Venezuela. Latin American Economic Review.

[18] Bjerkholt O., Niculescu I. (2004). Rules-based Fiscal Policy in Emerging Markets.

[19] Villafuerte M., Lopez-Murphy P. (2009). IMF Working Paper No. 10/28.

[20] Fuinhas J.A., Marques A. C. Rentierism (2013). Energy and economic growth: the case of Algeria and Egypt (1965–2010). Energy Pol..

[21] Alshehry A.S., Belloumi M. (2015). Energy consumption, carbon dioxide emissions and economic growth: the case of Saudi Arabia. Renew. Sustain. Energy Rev..

[22] Malik M.Y., Latif K., Khan Z., Butt H.D., Hussain M., Nadeem M.A. (2020). Symmetric and asymmetric impact of oil price, FDI and economic growth on carbon emission in Pakistan: evidence from ARDL and non-linear ARDL approach. Sci. Total Environ..

[23] Ali M., Tursoy T., Samour A., Moyo D., Konneh A. (2022). Testing the impact of the gold price, oil price, and renewable energy on carbon emissions in South Africa: novel evidence from bootstrap ARDL and NARDL approaches. Resour. Pol..

[24] Constantinos K., Eleni Z., Nikolaos S., Bantis D. (2019). Greenhouse gas emissions–crude oil prices: an empirical investigation in a nonlinear framework. Environ. Dev. Sustain..

[25] Cong Y., Freedman M. (2011). Corporate governance and environmental performance and disclosures. Adv. Account..

[26] Elsayih J., Datt R., Tang Q. (2021). Corporate governance and carbon emissions performance: empirical evidence from Australia. Australas. J. Environ. Manag..

[27] Elsayih J., Tang Q., Lan Y.-C. (2018). Corporate governance and carbon transparency: Australian experience. Account. Res. J..

[28] Liao L., Luo L., Tang Q. (2015). Gender diversity, board independence, environmental committee and greenhouse gas disclosure. Br. Account. Rev..

[29] Nuber C., Velte P. (2021). Board gender diversity and carbon emissions: European evidence on curvilinear relationships and critical mass. Bus. Strat. Environ..

[30] Tang Q., Luo L. (2016). Corporate ecological transparency: theories and empirical evidence. Asian Rev. Account..

[31] Tauringana V., Chithambo L. (2015). The effect of DEFRA guidance on greenhouse gas disclosure. Br. Account. Rev..

[32] Guthrie J., Parker L.D. (1990). Corporate social disclosure practice: a comparative international analysis. Adv. Publ. Interest Account..

[33] Ansoff H.I. (1965).

[34] Dowling J., Pfeffer J. (1975). Organizational legitimacy: social values and organizational behavior. Pac. Socio Rev..

[35] Deegan C. (2002). Introduction: the legitimising effect of social and environmental disclosures–a theoretical foundation. Account Audit. Account. J..

[36] Matthews M. (1993).

[37] Patten D.M. (1991). Exposure, legitimacy, and social disclosure. J. Account. Publ. Pol..

[38] Luo L., Tang Q., Peng J. (2018). The direct and moderating effects of power distance on carbon transparency: an international investigation of cultural value and corporate social responsibility. Bus. Strat. Environ..

[39] Luo L. (2019). The influence of institutional contexts on the relationship between voluntary carbon disclosure and carbon emission performance. Account. Finance.

[40] Hörisch J., Schaltegger S., Freeman R.E. (2020). Integrating stakeholder theory and sustainability accounting: a conceptual synthesis. J. Clean. Prod..

[41] Cotter J., Najah M.M. (2012). Institutional investor influence on global climate change disclosure practices. Aust. J. Manag..

[42] Ieng Chu C., Chatterjee B., Brown A. (2012). The current status of greenhouse gas reporting by Chinese companies: a test of legitimacy theory. Manag. Audit J..

[43] Tang Q., Luo L. (2014). Carbon management systems and carbon mitigation. Aust. Account. Rev..

[44] Datt R., Luo L., Tang Q., Mallik G. (2018). An international study of determinants of voluntary carbon assurance. J. Int. Account. Res..

[45] Stern N.H. (2006).

[46] Apergis N., Payne J.E. (2009). CO2 emissions, energy usage, and output in Central America. Energy Pol..

[47] Abumunshar M., Aga M., Samour A. (2020). Oil price, energy consumption, and CO2 emissions in Turkey. New evidence from a Bootstrap ARDL Test. Energies.

[48] Lardic S., Mignon V. (2006). The impact of oil prices on GDP in European countries: an empirical investigation based on asymmetric cointegration. Energy Pol..

[49] Rafiq S., Salim R., Bloch H. (2009). Impact of crude oil price volatility on economic activities: an empirical investigation in the Thai economy. Resour. Pol..

[50] Gorus M.S., Ozgur O., Develi A. (2019). The relationship between oil prices, oil imports and income level in Turkey: evidence from Fourier approximation. OPEC Energy Review.

[51] Rahmana S., Serletisb A. (2019). Oil prices and the stock markets: evidence from high frequency data. Energy J..

[52] Soytas U., Sari R., Ewing B.T. (2007). Energy consumption, income, and carbon emissions in the United States. Ecol. Econ..

[53] Stern D.I. (2000). A multivariate cointegration analysis of the role of energy in the US macroeconomy. Energy Econ..

[54] Umar M., Mirza N., Hasnaoui J.A., Rochoń M.P. (2022). The nexus of carbon emissions, oil price volatility, and human capital efficiency. Resour. Pol..

[55] Wang Y., Xiang E., Ruan W., Hu W. (2017). International oil price uncertainty and corporate investment: evidence from China's emerging and transition economy. Energy Econ..

[56] Chen X., Li Y., Xiao J., Wen F. (2020). Oil shocks, competition, and corporate investment: evidence from China. Energy Econ..

[57] Shen T., Hu R., Hu P., Tao Z. (2023). Decoupling between economic growth and carbon emissions: based on four major regions in China. Int. J. Environ. Res. Publ. Health.

[58] Maghyereh A., Abdoh H. (2020). Asymmetric effects of oil price uncertainty on corporate investment. Energy Econ..

[59] Saboori B., Al-Mulali U., Bin Baba M., Mohammed A.H. (2016). Oil-induced environmental Kuznets curve in organization of petroleum exporting countries (OPEC). Int. J. Green Energy.

[60] Fan H., Tang Q., Pan L. (2021). An international study of carbon information asymmetry and independent carbon assurance. Br. Account. Rev..

[61] He R., Luo L., Shamsuddin A., Tang Q. (2022). The value relevance of corporate investment in carbon abatement: the influence of national climate policy. Eur. Account. Rev..

[62] Xing X., Cong Y., Wang Y., Wang X. (2023). The impact of COVID-19 and war in Ukraine on energy prices of oil and natural gas. Sustainability.

[63] Elsayih J., Datt R., Hamid A. (2020). CEO characteristics: do they matter for carbon performance? An empirical investigation of Australian firms. Soc. Responsib. J..

[64] Luo L., Tang Q. (2014). Does voluntary carbon disclosure reflect underlying carbon performance?. J. Contemp. Account. Econ..

[65] Hasan M.M., Asad S., Wong J.B. (2022). Oil price uncertainty and corporate debt maturity structure. Finance Res. Lett..

[66] Luo L., Tang Q. (2022). The real effects of ESG reporting and GRI standards on carbon mitigation: international evidence. Bus. Strat. Environ..

[67] Bui B., Moses O., Houqe M.N. (2020). Carbon disclosure, emission intensity and cost of equity capital: multi‐country evidence. Account. Finance.

[68] Datt R., Luo L., Tang Q. (2020). Corporate choice of providers of voluntary carbon assurance. Int. J. Audit..

[69] Griffin P.A., Lont D.H., Sun E.Y. (2017). The relevance to investors of greenhouse gas emission disclosures. Contemp. Account. Res..

[70] Luo L., Tang Q. (2016). Does national culture influence corporate carbon disclosure propensity?. J. Int. Account. Res..

[71] Hair J., Black W., Babin B., Anderson R. (2015).

[72] Moussa T., Allam A., Elbanna S., Bani‐Mustafa A. (2020). Can board environmental orientation improve US firms' carbon performance? The mediating role of carbon strategy. Bus. Strat. Environ..

[73] Mensah I.A., Sun M., Gao C., Omari-Sasu A.Y., Zhu D., Ampimah B.C., Quarcoo A. (2019). Analysis on the nexus of economic growth, fossil fuel energy consumption, CO2 emissions and oil price in Africa based on a PMG panel ARDL approach. J. Clean. Prod..

[74] Katircioglu S. (2017). Investigating the role of oil prices in the conventional EKC model: evidence from Turkey. Asian Econ. Financ. Rev..

[75] Zhu B., Xu C., Wang P., Zhang L. (2022). How does internal carbon pricing affect corporate environmental performance?. J. Bus. Res..

[76] Heckman J.J. (1979). Sample selection bias as a specification error. Econometrica: J. Econom. Soc..

[77] Elsayih J., Datt R., Tang Q., Hamid A., Varua M.E. (2023). Exploring the determinants of carbon management system quality: the role of corporate governance and climate risks and opportunities. Account. Finance.

[78] Zheng S., Zhang Q., Zhang P. (2023). Can customer concentration affect corporate ESG performance?. Finance Res. Lett..

[79] Feng J., Yuan Y. (2024). Green investors and corporate ESG performance: evidence from China. Finance Res. Lett..

[80] Atif M., Alam M.S., Islam M.S. (2022). Firm-level energy and carbon performance: does sustainable investment matter?. Bus. Strat. Environ..

[81] Phan D.H.B., Tran V.T., Tee C.M., Nguyen D.T. (2021). Oil price uncertainty, CSR and institutional quality: a cross-country evidence. Energy Econ..

[82] Yang B., An H., Song X. (2024). Oil price uncertainty and corporate inefficient investment: evidence from China. N. Am. J. Econ. Finance.

[83] Safiullah M., Alam M.S., Islam M.S. (2022). Do all institutional investors care about corporate carbon emissions?. Energy Econ..

[84] Bui B., Houqe M.N., Zaman M. (2021). Climate change mitigation: carbon assurance and reporting integrity. Bus. Strat. Environ..

